# On-Site Physiotherapy in Emergency Department Patients Presenting with Nonspecific Low Back Pain: A Randomized Controlled Trial

**DOI:** 10.3390/jcm13113149

**Published:** 2024-05-27

**Authors:** Leon Chrobok, Tanguy Espejo, Henk B. Riedel, Joris Kirchberger, Jan-Arie Overberg, Florina Felber, Guido Perrot, Christian H. Nickel, Roland Bingisser

**Affiliations:** 1 Emergency Department, University Hospital, 4031 Basel, Switzerland; leon.chrobok@usb.ch (L.C.); tanguy.espejo@usb.ch (T.E.); henk.riedel@usb.ch (H.B.R.); florinalorena.felber@usb.ch (F.F.); christian.nickel@usb.ch (C.H.N.); 2 Department of Therapies, University Hospital, 4031 Basel, Switzerland; joris.kirchberger@usb.ch (J.K.); johannes.overberg@usb.ch (J.-A.O.); guido.perrot@usb.ch (G.P.)

**Keywords:** randomized controlled trial, physiotherapy, nonspecific low back pain, feasibility, satisfaction, disability, emergency department

## Abstract

**Background:** There is a high incidence of nonspecific Low Back Pain (LBP) in patients visiting Emergency Departments (EDs), but there is a lack of knowledge regarding emergency physiotherapy for LBP. The effect of on-site physiotherapy in these patients was therefore never demonstrated. We assessed short-term outcomes, feasibility and patient satisfaction with physiotherapy in ED patients presenting with nonspecific LBP. **Methods:** A block-randomized, controlled, open-label trial with a follow-up of 42 days. Patients aged 18 years or older presenting to an ED with nonspecific LBP were prospectively enrolled. Both groups received the same booklet with written information on LBP management and exercises. Patients in the intervention group were given additional instructions by a certified physiotherapist. **Results:** We included 86 patients in the primary analysis. The median age was 40, and 40.7% were female. At day 7, the median Oswestry Disability Index (ODI) was 2 points lower in the intervention group compared to the control group, which was not statistically significant. There was no between-group difference in pain at day 7. Patients who received physiotherapy felt significantly more confident with the exercises they were taught (*p* = 0.004, effect size = 0.3 [95% CI 0.1 to 0.5]). **Conclusions:** On-site physiotherapy in ED patients presenting with nonspecific low back pain is associated with higher patient satisfaction, compared to standard of care. The effect of physiotherapy was small, with only minimal improvement in disability, but without a reduction in pain. Despite the very small effect size, physiotherapeutic interventions should be investigated in larger cohorts with an extended intervention including patient education, exercises, and other physiotherapeutic modalities.

## 1. Introduction

More than a third of all adults have suffered from lower back pain (LBP) in the past year [1], making LBP the leading cause of years lived with disability (YLDs) [2]. Nonspecific LBP, defined as low back pain without any clear association with a pathoanatomical cause, accounts for the vast majority of low back pain [3]. In emergency departments (EDs), LBP accounts for more than four percent of all presentations, according to a meta-analysis pooling multiple prevalence studies [4].

As there are no specific guidelines for the management of nonspecific LBP in EDs, a wide variation in the management of these patients has been described [5,6]. However, guidelines originating in primary care could be applied to the ED setting [5]. These guidelines recommend “early” physiotherapy [7,8,9], and an adherence to these recommendations seem to improve pain, disability, total cost of physiotherapy [10], and a reduction in the utilization of medical resources [11]. The timing of physiotherapy for patients suffering from acute LBP was examined in a systematic review, showing that early intervention, defined as within 30 days, resulted in a lower use of resources, and might have the potential to prevent recurrences and chronicity [12]. One study suggested that commencing physiotherapy within 3 days was associated with the lowest health service use, and with lower rates of advanced imaging and opioid medication, compared to physiotherapy after this timepoint [13]. In the ED setting, a retrospective observational study showed that patients with nontraumatic neck and back pain experienced lower levels of disability and pain intensity if they received physiotherapy during their ED visit, compared to those who received outpatient physiotherapy at a median of 34 days after presentation [14]. Furthermore, from the perspective of patients with acute LBP, direct access to physiotherapy in the ED was rated as beneficial [15]. In addition, a randomized controlled trial (RCT) demonstrated that direct access to physiotherapy in the ED for patients with musculoskeletal disorders resulted in a greater reduction in pain compared to no physiotherapy, and led to a reduced use of medical resources [16].

To our knowledge, there is only one published RCT investigating the effect of early physiotherapy intervention in patients with LBP in an Accident and Emergency Department in Hong Kong. In this study, physiotherapy was shown to reduce pain and increase patient satisfaction, but only within the first month [17].

In summary, there is uncertainty about the impact of on-site physiotherapy in the ED setting [18]. To fill this knowledge gap, the aims of this randomized controlled trial were to investigate outcomes such as disability, feasibility and patient satisfaction with physiotherapy during ED work-up in patients presenting with nonspecific LBP [14,17,18,19,20].

## 2. Materials and Methods

### 2.1. Study Design and Setting 

This was a monocentric, block-randomized, controlled, open-label, parallel-group trial. The trial was conducted in the ED of the University Hospital Basel, in Northwestern Switzerland, between 3 January 2022 and 30 June 2023. The University Hospital Basel is an urban tertiary care center with an ED census of more than 55,000 patient visits annually. The study protocol was approved by the local Ethics Committee (“EKNZ: Ethikkommission Nordwest- und Zentralschweiz”, project N° 2021-02166) and can be accessed there. The date of ethical approval was 7 December 2021. The study was prospectively registered on the clinicaltrials.gov website (study N° NCT05156957). Our study is reported in accordance with the Consolidated Standards of Reporting Trials Guidelines (Table S1) [21].

### 2.2. Selection of Participants

Patients suffering from nonspecific LBP aged 18 or older who presented to the ED were eligible for the study. The World Health Organization defines LBP as “pain between the lower edge of the ribs and the buttock” [22]. In our study, nonspecific LBP was defined as LBP in the absence of major trauma, fracture leading to immobilization, severe or progressive sensory alteration or weakness, bladder or bowel dysfunction, neurological deficit on physical examination or severe chronic disease, such as metastatic cancer, severe renal insufficiency and palliative care needs. Exclusion criteria were prior enrollment in this trial, inpatient disposition after ED work-up (as these patients would benefit from physiotherapy during the hospitalization), epidural steroid injections in the last 3 months, inability or contraindications to undergo the intervention or to follow the study procedures, e.g., due to certain neurological disorders, language problems, psychological disorders, cognitive impairment or physical inability. Patients were recruited during weekdays from 8 AM to 5 PM in the presence of investigators.

### 2.3. Randomization

A block randomization was performed. Randomization was conducted using R (version 4.3.1) [23] by randomizing each week in advance. It was randomized whether the physiotherapist was present in the morning or in the afternoon during each work-week. Therefore, the physiotherapist was present for 50% of the inclusion period, on weekdays either in the morning from 8 AM to 12 PM or in the afternoon from 1 PM to 5 PM. Patients were included in the intervention group when the physiotherapist was present, with a 1:1 allocation ratio. Eligible patients were approached by the study team. The blinding of the patients was achieved by giving verbal and written informed consent in the ED without informing the patients about the group allocation or the exact extent of the intervention. After the 7-day follow-up, patients were given a second written informed consent that included information about group allocation and the intervention. The study team and the physiotherapists were not blinded.

### 2.4. Groups

#### 2.4.1. Intervention

**Figure 1:**
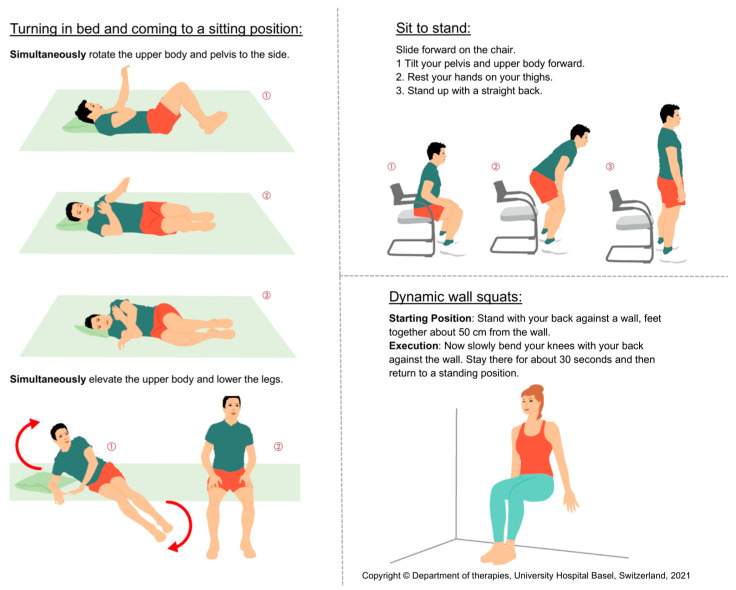
Illustration of the three exercises. This is an extract from the booklet that was provided to each participant at baseline. The three exercises (turning in bed and coming to a sitting position, sit to stand and dynamic wall squats) are each described with written and pictorial explanations.

The intervention group received the following intervention from a physiotherapist in the ED: an initial history regarding pain was taken, red flags were checked, questionnaires previously completed by the patient (Oswestry Disability Index (ODI) and STarT-Back Screening tool) were reviewed and a back performance test using the Back Performance Scale (BPS) was performed to provide the physiotherapist with a general overview of the patient’s back pain. The patient was then given information about the expected course of the condition and self-management instructions regarding back-friendly behaviors: minimize bed rest, stay active and walk, perform activities while sitting or standing, change position regularly, avoid prolonged sitting or standing and pacing (respecting the pain and finding the right balance between activity and rest to avoid worsening symptoms due to overexertion). In addition, three exercises for daily self-guided therapy were shown and explained to the patient. These three exercises were turning in bed and coming to a sitting position, sit to stand and dynamic wall squats. Figure 1 displays these exercises. Finally, the patient received a written booklet with all the instructions and the explanations for the exercises mentioned so far. The physiotherapist encouraged the patient to follow the recommendations. The intervention was conducted face-to-face at the bedside in an ED examination room and took an average of 19 min. The selection of the intervention is based on the scientific evidence indicating that patient education, behavioral instructions and active movement exercises taught by a professional are beneficial for patients with LBP [24,25,26,27,28,29].

Over the course of the study, two different physiotherapists administered the intervention. Both have degrees in physical therapy, where the management and treatment of back pain is an integral part of the curriculum.

The intervention is reported in accordance with the Template for Intervention Description and Replication (TIDieR) Guidelines (Table S2) [30].

#### 2.4.2. Control

Patients in the control group did not receive any physiotherapy intervention. They received the same booklet as the intervention group containing written information on the expected course of the condition, written instructions on self-management and written instructions on exercises for daily self-guided therapy. The booklet was given to patients by the non-physiotherapy study team (study physicians with less than 1 year of clinical experience). The study physicians only mentioned that the booklet contained behavioral recommendations and exercises, but did not explain or demonstrate them. The control group “intervention” took an average of 2 min.

### 2.5. Measurements

For all patients included, baseline data involving demographics (date of birth, gender, ethnicity, height, weight, occupation), medical history and numeric rating scale (NRS) of LBP were collected in the ED. To obtain an overview of the physical condition of the patients, the physical activity vital sign was used, which indicates the total number of minutes of physical activity per week [31]. This included quantifying the average number of days per week of moderate to strenuous physical activity and the number of minutes of physical activity on those days. Pain medication use was measured by asking patients about the duration of pain medication intake in days. The ODI, an instrument for measuring the functional status of patients with LBP and associated disability, was obtained. ODI was validated in German and showed a good reliability [32]. The ODI consists of 10 questions, each with 6 possible answers from 0 to 5. The questions relate to the disability caused by back pain in terms of pain intensity, pain during different physical activities, sleep, sex life, social life and travel. A higher score indicates greater disability due to back pain [33]. In addition, the STarT-Back Screening tool, an instrument for patients with nonspecific LBP, was used by applying a questionnaire [34]. Nine questions on physical and psychological risk factors are used to assess the psychosocial aspect of back pain and therefore the risk of pain chronification. A maximum of one point is awarded for each question, resulting in a possible total score of 0 to 9. A score of 3 or fewer points indicates a low risk of pain chronification. If the score is 4 points or more, the results of questions 5 to 9 are added together to form a subscore. If the subscore is 3 or fewer points, this indicates a medium risk of chronification and if it is 4 or more points, this indicates a high risk of chronification. This allows patients to be categorized into risk categories. The tool has been validated in German [35,36]. Information about the Emergency Severity Index (ESI) [37] and vital signs at admission (heart rate, systolic and diastolic blood pressure, oxygen saturation) were collected from the electronic clinical information system (ISMed^®^ byProtec-Data, Boswil, Switzerland). 

Furthermore, the Back Performance Scale (BPS), a measurement tool to evaluate the physical performance and functional capacity of participants with low back pain, was assessed during the physiotherapeutic intervention. The BPS includes 5 physical tests (sock test, pick-up test, roll-up test, fingertip-to-floor test and lift test), each with a score from 0 (no physical impairment) to 3 (severe physical impairment), giving a total score from 0 to 15 [38]. The feasibility of the physiotherapeutic intervention was assessed using a questionnaire completed by the physiotherapist on the day of inclusion after the intervention. The questionnaire, consisting of six questions, was designed by the authors of the study to assess feasibility from the physiotherapists’ perspective. Each question had a scale of 1 (=no, strongly disagree) to 6 (=yes, strongly agree) for possible answers, inspired by the Swiss grading system.

Between 7 and a maximum of 14 days after inclusion, patients of both groups had an appointment with a physiotherapist, which represents the first follow-up visit and not a physiotherapeutic intervention. Patients were seen by the same physiotherapist who was present at the time of enrollment. If the physiotherapist was unavailable, a second physiotherapist covered for them, if available. First, each patient completed a questionnaire containing 6 different questions about their satisfaction with their ED work-up. The questionnaire was designed by the authors of the study. Each question could be answered with a score from 0 (not at all) to 10 (very much). They were then interviewed about the utilization of medical resources by asking if they had seen a physiotherapist, an emergency department, a general practitioner, a specialist, been hospitalized or received imaging (X-ray, CT or MRI) since the last contact. In addition, NRS of LBP, pain medication use, BPS, ODI and STarT-Back Screening Tool were assessed. The ability to work was assessed by asking patients if they were currently working. Also, adherence to therapy recommendations was assessed by asking if they could avoid bed rest, change position regularly, adhere to pacing, take walks, and perform the three exercises provided (turning in bed and coming to a sitting position, sit to stand, and dynamic wall squats). At the end of the follow-up, the physiotherapist may or may not have recommended a physiotherapy intervention for the patient, depending on their assessment of the patient’s condition.

In addition, two follow-up telephone interviews were conducted by the study physicians on day 21 and day 42 after inclusion. In both interviews, information about ability to work, utilization of medical resources, NRS of LBP and pain medication use were obtained. On day 21, the ODI questionnaire, and on day 42, the ODI and STarT-Back questionnaire were applied. 

### 2.6. Outcomes

The primary outcome was the difference in the ODI between groups, as assessed after 7 days. Secondary outcomes were the use of medical resources, overall improvement of pain (NRS), pain medication use, ability to work, risk of pain chronification measured using the STarT-Back Screening Tool and the ODI over the whole follow-up period. Feasibility of the physiotherapeutic intervention at baseline, patient satisfaction with their ED work-up and the adherence to therapy recommendations after 7 days were also secondary outcomes.

### 2.7. Statistical Analysis

#### 2.7.1. Sample Size Calculation

For our sample size estimation, we considered studies that used the ODI to derive the estimated benefit of our intervention [32,39,40,41]. We chose a mean difference of 5 points, as this is slightly below the number usually defined as the minimum clinically important difference [42,43], with a standard deviation of 10. With these values from the literature, an effect size of 0.5 was calculated using Cohen’s d [44]. With the given effect size, 80% power and α = 0.05, we calculated a required population of 64 patients per group. Assuming a loss of 10% of participants due to drop-out, loss to follow-up or secondary exclusion (due to hospitalization for example), we aimed to recruit 70 participants per group, 140 patients in total.

#### 2.7.2. Primary Data Analysis 

An intention-to-treat analysis was conducted. Missing data were handled with available case analyses. Descriptive statistics are expressed as counts and percentages or as medians with interquartile ranges (IQR). The data were tested for normal distribution, and differences were tested using a Student’s *t*-test for normally distributed data and using a Wilcoxon test for non-normally distributed data. The significance level is two-sided, with a level of α = 0.05. In order to quantify the magnitude of the effect from the Wilcoxon test, we used the Wilcoxon effect size [45] from the *rstatix* package in R [23] to calculate the effect size r. The interpretation values for the effect size r are based on the literature [45,46]: 0.10–<0.3 (small effect), 0.30–<0.5 (moderate effect) and ≥0.5 (large effect). Pain intensity was assessed using the NRS and a minimum clinically significant difference of 2 points was selected, which was derived from the literature [47,48]. All analyses were performed using R (version 4.3.1) [23].

## 3. Results

**Figure 2:**
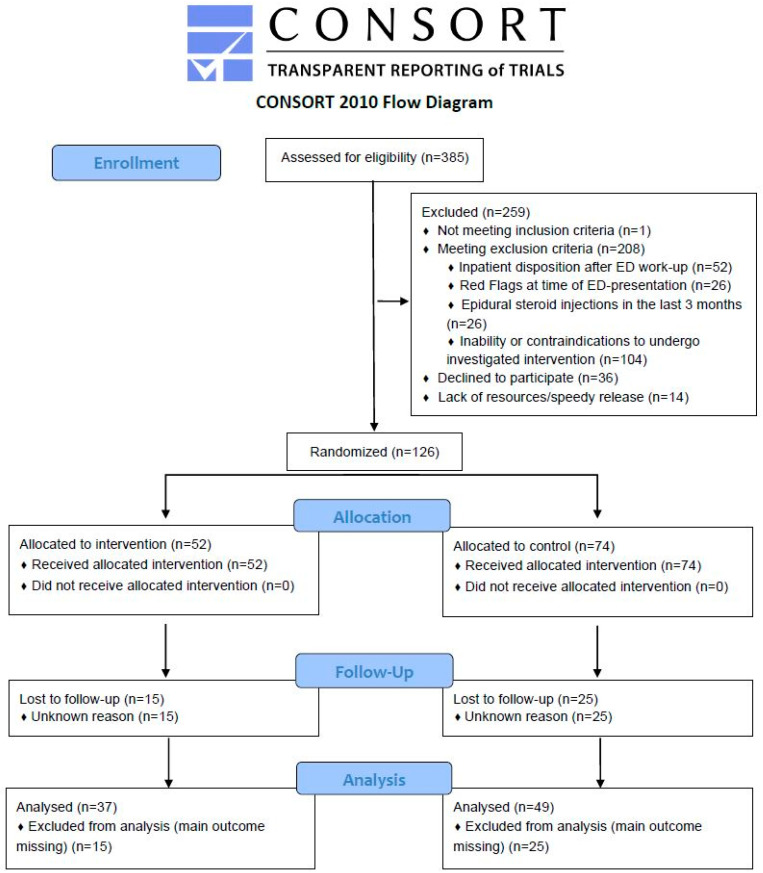
Flow chart of the study population. The chart displays recruitment procedure of emergency department patients aged 18 or older presenting with nonspecific low back pain.

A total of 385 patients were screened for eligibility between January 2022 and June 2023. Overall, 259 patients were excluded, resulting in 126 patients being randomized. Fifty-two patients were allocated to the intervention group and received the intervention. Among these, 15 patients were lost to follow-up, leaving 37 patients for the analysis. A total of 74 patients were assigned to the control group and 25 of them were lost to follow-up, resulting in 49 patients analyzed in the control group (details in Figure 2).

The baseline characteristics were similar between patients from the control and intervention groups. The median age of participants was 40 years (IQR, 32–52) and 40.7% of the 86 patients were female. The median body mass index (BMI) was 25 (IQR, 23–29), the median physical activity vital sign was 150 min per week (IQR, 5.5–300) and 69.8% of the 86 participants took pain medication recently before the initial consultation. No participant was triaged to ESI level 1, 11 (12.8%) patients to ESI level 2, 28 (32.6%) to ESI level 3, 45 (52.3%) to ESI level 4 and 1 (1.2%) to ESI level 5. Median pain on the NRS was 7.5 (IQR, 5–8) on a scale from zero for no pain at all to 10 for maximum pain. The median score on the STarT-Back-Questionnaire on the day of consultation was 5 (IQR, 4–6) and the median ODI score was 24 (IQR, 16–33) (Table 1).

**Figure 3:**
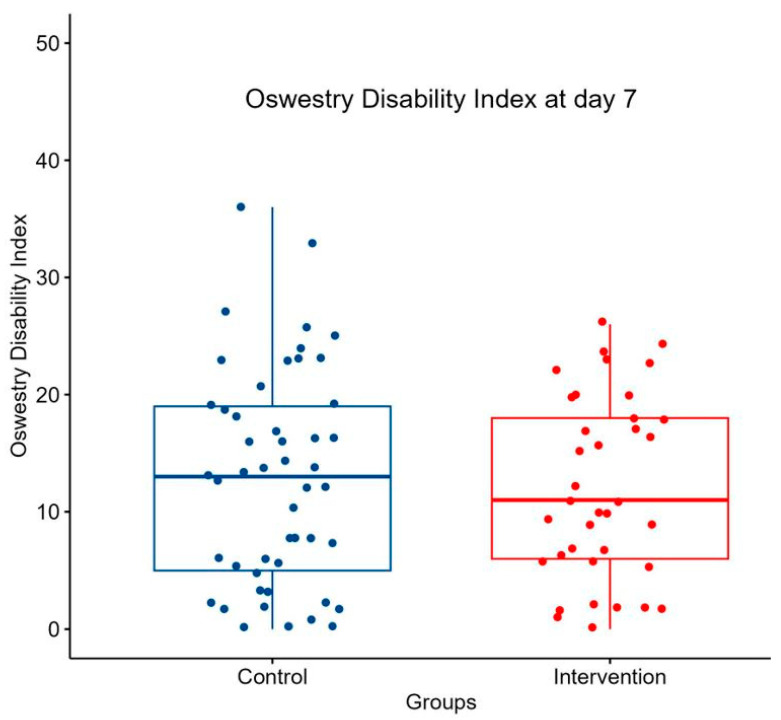
Boxplot of the Oswestry Disability Index at day 7. Boxplot comparing ODI at day 7 between control group and intervention group. Single points symbolize patients. Box contains Interquartile Range (IQR) and horizontal line inside the box indicates the median.

The median ODI at day 7 in the control group was 13 (IQR, 5–19), whereas it was 11 (IQR, 6–18) in the intervention group (as demonstrated in Figure 3 and Table 2). The Wilcoxon test showed that the difference between the two groups was not significant (*p* = 0.854, effect size r = 0.0198 [95% CI 0.003 to 0.25]).

There was no significant difference between the two groups after 7 days in terms of the STarT-Back Screening Tool, pain on NRS, pain medication use and ability to work (shown in Table 2).

The results of the questionnaire on the feasibility of the physiotherapist’s intervention on day zero are shown in Table 3. 

**Figure 4:**
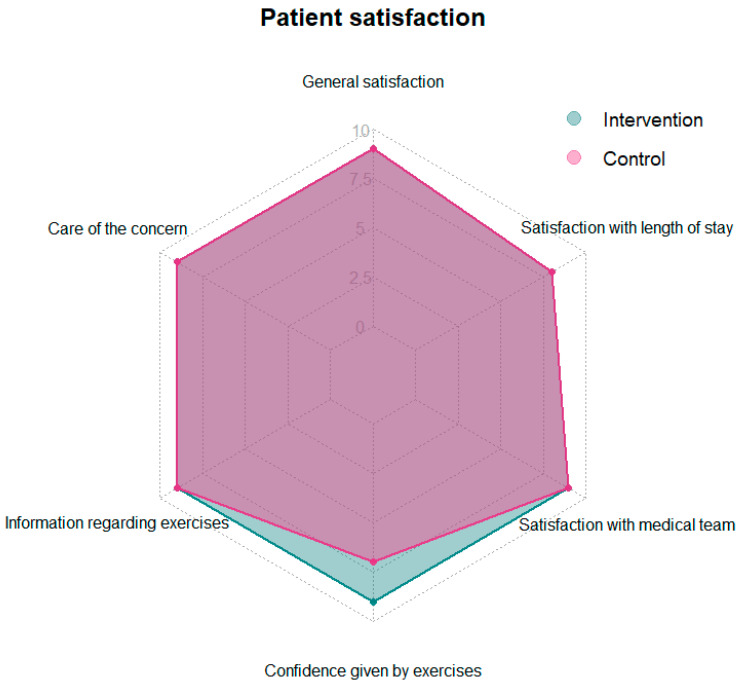
Radar chart of the patient satisfaction at day 7. Patients completed a questionnaire about their satisfaction with their ED visit. Answers were recorded on a Numeric Rating Scale (NRS) from 0 (=not at all) to 10 (=very much). Displayer values are medians.

Comparing the two groups in terms of patient satisfaction on day 7, it was found that patients in the intervention group felt more confident with the exercises instructed by a physiotherapist, with a median of 9 (IQR, 8–9.25) on the questionnaire. Patients in the control group had a median score of 7 (IQR, 3–9) on the questionnaire in terms of confidence from the exercises. The Wilcoxon test showed that the difference was significant (*p* = 0.004, effect size r = 0.310 [95% CI 0.1 to 0.5] (See Table 4 and Figure 4). For the remaining patient satisfaction questions, there was no difference between the groups.

Adherence to the therapy recommendations was assessed in both groups during the first follow-up. As shown in Table 5, a larger proportion of patients in the intervention group followed each therapy recommendation compared to patients in the control group. Statistically significant results included more patients in the intervention group avoiding bed rest (*p* = 0.005), adhering to pacing recommendations (*p* = 0.033), performing the “turning in bed and coming to a sitting position” exercise (*p* = 0.009) and the “squats standing in front of a wall” exercise (*p* = 0.003) than in the control group. 

Concerning the utilization of medical resources, there was no significant difference between the groups at each of the three follow-ups (Table A1). 

Comparing the outcomes on day 21 (follow-up 2), there was no significant difference between the groups in terms of ODI, pain on NRS, pain medication use and the ability to work (Table A2). 

Similarly, comparing the outcomes at day 42 (follow-up 3), there was no significant difference between the groups in terms of STarT-Back Screening Tool, pain on NRS, pain medication use and the ability to work (Table A3). The median ODI on day 42 in the control group was 4 (IQR, 0–16), whereas the median ODI on day 42 in the intervention group was 2 (IQR, 0.75–6.75). In the intervention group, there were two patients in the “moderate disability” group on day 42, while in the control group there were eight patients in this group. There were no differences between the two groups in the other subcategories of the ODI (Table A3).

As shown in Figure 2, 40 of the original 126 randomized patients were lost to follow-up. The baseline characteristics of these 40 lost to follow-up patients are compared with those of the 86 patients analyzed in Table A4. The two groups are very similar. There are no differences in age, BMI, pain medication use, pain on NRS, STarT-Back Screening tool and ODI. Patients in the lost to follow-up group were more physically active with a median of 270 min in the physical activity vital sign compared to patients in the analyzed group with a median of 150 min.

We observed no harm or unintended effects in either the intervention group or the control group.

## 4. Discussion

The main result of this trial was that patients with on-site physiotherapy had a two-point lower ODI, corresponding to a lower level of disability at day 7. The difference was statistically not significant (*p* = 0.85, effect size r = 0.02) and did not meet the predefined clinically important difference of 5 points. This corroborates findings in primary care showing a modest improvement, not reaching the clinically important difference as compared with usual care [49]. 

Secondly, the intervention was associated with less moderate disability, as shown by a difference in ODI categories at day 42. Despite the small number of observations, the implementation of ED-based physiotherapy could potentially have an impact on disability in nonspecific acute LBP. A non-randomized study previously showed that ED-initiated physiotherapy can improve disability compared with usual care [50]. Patients were assigned to physiotherapy intervention at the discretion of the treating physician, which might have biased treatment allocation and led to unequal baseline characteristics. 

Thirdly, the participants in the intervention group showed substantially higher adherence to the therapy recommendations compared to those in the control group. Behavioral recommendations for nonspecific LBP were implemented up to 28 percentage points more often when patients received a physiotherapy intervention. Furthermore, exercise adherence was up to 36 percentage points higher when patients received exercise instruction as part of the intervention. This indicates that ED-based physiotherapy might improve treatment adherence. In the long term, this may be beneficial for patients to prevent recurrent episodes of LBP. On the other hand, patients in the control group were adherent to most of the behavioral recommendations and exercises more than 50% of the time, even though they only received written instructions as the standard of care.

For all other outcomes, there were no differences between the groups (pain on NRS, STarT-Back Screening Tool, pain medication use, utilization of medical resources and ability to work). 

Pain was reduced to about half of the baseline level after 7 days in both groups. This suggests that in our cohort of patients seeking attention in the ED, episodes of LBP were rather short-lived, and physiotherapy did not seem to provide additional pain relief over standard of care. However, patients in both groups were given a booklet with information on helpful exercises and general behavior. 

This standard of care might not be implemented in all EDs and could also have contributed to the early reduction in pain. These findings are at contrast with those of a retrospective study with a less stringent standard of care [14] and with the only published RCT, in which pain relief was better in the physiotherapy intervention group. However, important differences to our study must be noted, e.g., that only the physiotherapy group received mobility training, 15 min of interferential therapy and strengthening exercises, whereas the control group only received walking training and walking aids [17]. Patients diagnosed with nonspecific LBP, however, should always receive education regarding their condition, back-friendly behavior, and strengthening exercises. This can be accomplished by providing written information containing all the necessary information.

Fourthly, patients were generally satisfied with the care they received in the ED, although the intervention group felt significantly more confident with the exercises taught by physiotherapists. Other studies have also shown that patients are more satisfied when they receive physiotherapy compared to the standard of care [15,17,51,52]. 

Finally, physiotherapists rated the feasibility of an intervention in the ED as very high. It can be concluded that this type of short and early intervention is feasible in a busy inner-city ED environment, supporting previous findings [53]. Retrospective data have also shown that physiotherapy is safe, does not lead to increased readmissions, and is associated with patient satisfaction [54].

The debate surrounding direct access to physiotherapy in emergency departments for patients presenting with musculoskeletal complaints, including LBP, is becoming increasingly relevant. In other European countries, such as France [55] and Italy [56], this issue is being addressed with considerable attention. To our knowledge, there are no larger emergency departments (EDs) in Switzerland with directly affiliated physiotherapy services. Therefore, the results of this study are important in defining the future role of physiotherapy in this type of care. However, further studies are necessary that consider an adequate control group and address the question of whether and how physiotherapists can support emergency physicians when treating nonspecific LBP [57].

### Limitations

The main limitation of our study was under-recruitment. Due to the end of funding and the drop-out rate of almost a third, the planned sample size of 140 patients could not be reached. As a result, we were only able to the analyze data of 86 patients. A comparison of baseline characteristics between the analyzed patients and the lost to follow-up patients showed that the two groups were very similar in almost all characteristics. The only differences were a slightly higher proportion of men in the lost to follow-up group and a higher level of physical activity, indicating less severe symptoms. Another possible explanation of this considerable loss to follow-up could be the acute and transient nature of acute LBP. In our study population, pain was halved after seven days, which is comparable to other cohorts [58]. For participants, early improvements could have reduced the motivation for further participation. 

Block randomization did not completely balance the two groups in terms of size, mainly due to the unavailability of physiotherapists. However, patient characteristics were balanced.

As this study was conducted in a Swiss ED, the study population consisted mainly of Caucasians of European descent. In addition, the Swiss healthcare system is well funded and we speculate that the Swiss population may have higher health expectations, resulting in a lower threshold for patients presenting to the ED with a medical problem. Therefore, the generalizability of our findings to other populations is limited.

It can further be assumed that in a real-life setting more patients might accept and subsequently benefit from physiotherapy. Both inclusion/exclusion criteria and the 42-day follow-up could have been barriers to participation, preventing a higher inclusion rate.

The patient satisfaction questionnaire was designed by the authors of the study, and is therefore not validated and represents a limitation.

However, the very small effect size regarding the primary outcome can be taken as important and new information for planning other trials. A recalculation of sample size showed that more than ten thousand would need to be included for a significant difference between intervention and standard of care. Our sample size of 86 patients is sufficient for a small effect size in the context of a pilot study [59]. Therefore, the presented trial could be interpreted as a pilot study on possible effect sizes in trials with a very high standard of care within the control group for future research. 

## 5. Conclusions

On-site physiotherapy in ED patients presenting with nonspecific low back pain is feasible and is associated with higher patient satisfaction, compared to standard of care. The effect of physiotherapy was small, with only a minimal improvement in disability using the ODI and no reduction in pain. Despite the very small effect size, physiotherapeutic interventions should be investigated in larger cohorts and with an extended intervention including patient education, exercises, and other physiotherapeutic modalities.

## Figures and Tables

**Table 1:** Baseline characteristics (at day 0).

	Overall (*n* = 86)	Control (*n* = 49)	Intervention (*n* = 37)
Age (in years), median [IQR]	40 [32, 52]	41 [32, 50]	38 [31, 58]
Female sex, *n* (%)	35 (40.7)	20 (40.8)	15 (40.5)
BMI (in kg/m^2^), median [IQR]	25 [23–29]	25 [23, 28]	26 [24, 29]
Physical Activity Vital Sign, median [IQR]	150 [5.5, 300]	160 [0, 320]	120 [21, 280]
Pain medication intake before consultation, *n* (%)	60 (69.8)	36 (73.5)	24 (64.9)
ESI level, *n* (%)			
1	0	0	0
2	11 (12.8)	7 (14.3)	4 (10.8)
3	28 (32.6)	15 (30.6)	13 (35.1)
4	45 (52.3)	25 (51.0)	20 (54.1)
5	1 (1.2)	1 (2.0)	0 (0)
Pain on NRS (0 to 10), median [IQR]	7.5 [5, 8]	8 [5, 9]	7 [4, 8]
STarT-G, median [IQR]	5 [4, 6]	4 [3, 5]	5 [4, 6]
STarT-G risk categories, *n* (%)			
Low risk	16 (18.6)	13 (26.5)	3 (8.1)
Intermediate risk	54 (62.8)	28 (57.1)	26 (70.3)
High risk	16 (18.6)	8 (16.3)	8 (21.6)
Oswestry Disability Index, median [IQR]	24 [16, 33]	24 [16, 33]	24 [17, 33]
Oswestry Disability Index categories, *n* (%)			
No Disability (ODI 0–4)	2 (2.3)	1 (2.0)	1 (2.7)
Mild disability (ODI 5–14)	17 (19.8)	11 (22.4)	6 (16.2)
Moderate disability (ODI 15–24)	26 (30.2)	14 (28.6)	12 (32.4)
Severe disability (ODI 25–34)	24 (27.9)	13 (26.5)	11 (29.7)
Completely disabled (ODI 35–50)	17 (19.8)	10 (20.4)	7 (18.9)

Data are reported as median [interquartile range] or *n* (%). Abbreviations: BMI = body mass index, ESI = emergency severity index, NRS = numeric rating scale, ODI = Oswestry Disability Index.

**Table 2:** Primary and secondary outcomes (7 days ± 7 days after inclusion).

	Control (*n* = 49)	Intervention (*n* = 37)	*p*-Value
Oswestry Disability Index, median [IQR]	13.00 [5.00, 19.00]	11.00 [6.00, 18.00]	0.854
Oswestry Disability Index categories, *n* (%)			0.633
No disability (ODI 0–4)	11 (22.4)	7 (18.9)	
Mild disability (ODI 5–14)	18 (36.7)	14 (37.8)	
Moderate disability (ODI 15–24)	15 (30.6)	15 (40.5)	
Severe disability (ODI 25–34)	4 (8.2)	1 (2.7)	
Completely disabled (ODI 35–50)	1 (2.0)	0 (0.0)	
STarT-Back Screening Tool, median [IQR]	5.00 [3.00, 6.00]	4.00 [3.00, 5.00]	0.326
STarT-Back risk categories, *n* (%)			0.438
Low risk	16 (32.7)	13 (35.1)	
Intermediate risk	21 (42.9)	19 (51.4)	
High risk	12 (24.5)	5 (13.5)	
Pain on NRS, median [IQR]	3.50 [2.00, 5.00]	3.00 [2.00, 4.25]	0.359
Pain medication use, *n* (%)	26 (53.1)	17 (45.9)	0.359
Duration in days of painkillers intake, mean {SD}	4.58 {2.80}	5.37 {1.64}	0.295
Returned to work, *n* (%)	31 (63.3)	20 (54.1)	0.606

Data are reported as median [interquartile range], *n* (%) or mean {standard deviation}. *p*-values were calculated using a significance level (alpha) of 0.05. Abbreviations: ODI = Oswestry Disability Index, NRS = numeric rating scale.

**Table 3:** Feasibility of the intervention (at day 0).

	Intervention (*n* = 37)
Number of interruptions during the intervention, *n* (%)	
0 (interruptions)	29 (78.4)
1 (interruptions)	8 (21.6)
Could the patient’s medical needs be met with the standardized physical therapy approach? *, median [IQR]	5.0 [4.0–6.0]
Could the patient’s psychosocial needs be met using the standardized physiotherapy approach? *, median [IQR]	5.0 [4.0–6.0]
Was the patient open to physiotherapeutic intervention in the emergency center? *, median [IQR]	6.0 [5.0–6.0]
Were there any disruptions in the organizational integration [patient scheduling, interruptions, transfers within the emergency care center, noise, or chaos] of the physiotherapy intervention in the emergency care center in this case? *, median [IQR]	1.0 [1.0–2.0]
From a physiotherapy point of view, does physiotherapy make sense in this case at this time? *, median [IQR]	6.0 [4.0–6.0]
Did the interprofessional collaboration on the part of physiotherapy work in terms of information exchange? *, median [IQR]	6.0 [5.0–6.0]

The questions were answered by the physiotherapist after performing the intervention at day 0. Data are reported as median [interquartile range] or *n* (%). * On a scale of 1 to 6: 1 = no, strongly disagree; 2 = no, disagree; 3 = no, slightly disagree; 4 = yes, slightly agree; 5 = yes, agree; 6 = yes, strongly agree.

**Table 4:** Patient satisfaction at day 7.

	Control (*n* = 49)	Intervention (*n* = 37)	*p*-Value
How satisfied were you with your visit to the emergency center? median [IQR]	9.00 [8.00, 10.00]	9.00 [8.00, 10.00]	0.844
How well was your concern taken care of? median [IQR]	9.00 [8.00, 10.00]	9.00 [8.00, 10.00]	0.294
How satisfied were you with the information provided regarding exercise behavior? median [IQR]	9.00 [7.00, 10.00]	9.00 [8.00, 10.00]	0.145
Did the exercises give you more confidence? median [IQR]	7.00 [3.00, 9.00]	9.00 [8.00, 9.25]	0.004
How satisfied were you with the medical care (without the study team) in the emergency center? median [IQR]	9.00 [8.00, 10.00]	9.00 [8.00, 10.00]	0.559
How do you estimate your overall length of stay in the emergency department? median [IQR]	8.00 [6.00, 10.00]	8.00 [5.00, 10.00]	0.343

Patients completed a questionnaire about their satisfaction with their ED visit. Answers were recorded on a Numeric Rating Scale (NRS) from 0 (= not at all) to 10 (= very much). Data are reported as median [interquartile range]. *p*-values were calculated using a significance level (alpha) of 0.05.

**Table 5:** Adherence to the therapy recommendations made on day 0 (assessed on day 7 ± 7 days after inclusion).

	Control (*n* = 49)	Intervention (*n* = 37)	*p*-Value
Avoiding bed rest, *n* (%)	37 (75.5)	37 (100)	0.005
Position changed regularly, *n* (%)	42 (85.7)	36 (97.3)	0.089
Adhere to pacing, *n* (%)	27 (55.1)	31 (83.8)	0.033
Performed walks, *n* (%)	40 (81.6)	35 (94.6)	0.155
Performed: Turning in bed and coming to a sitting position, *n* (%)	25 (51)	31 (83.8)	0.009
Performed: Sit to stand exercise, *n*(%)	28 (57.1)	31 (83.8)	0.055
Performed: Squats standing in front of a wall, *n* (%)	22 (44.9)	30 (81.1)	0.003

Data are reported as *n* (%). *p*-values were calculated using a significance level (alpha) of 0.05.

## Data Availability

The dataset is not available due to ethical restrictions. Upon request, the release of the dataset can be requested from the responsible ethics committee.

## Supplementary Materials

The following supporting information can be downloaded at: https://www.mdpi.com/article/10.3390/jcm13113149/s1, Table S1: CONSORT 2010 checklist of information to include when reporting a randomised trial; Table S2: TIDieR Checklist—Information to include when describing an intervention and the location of the information.

## Appendix A

**Table A1:** Utilization of medical resources.

	Control (*n* = 49)	Intervention (*n* = 37)	*p*-Value
**Follow-up day 7:**			
Physiotherapy in the last 7 days (other than intervention at day 0), *n* (%)	12 (24.5)	8 (21.6)	0.957
ED visit in the last 7 days (other than visit at day 0), *n* (%)	3 (6.1)	3 (8.1)	1.000
GP consultation in the last 7 days, *n* (%)	15 (30.6)	16 (43.2)	0.327
Specialist visit in the last 7 days, *n* (%)	7 (14.3)	3 (8.1)	0.586
Hospitalization in the last 7 days, *n* (%)	1 (2.0)	1 (2.7)	1.000
Imaging in the last 7 days, *n* (%)	6 (12.2)	2 (5.4)	0.229
**Follow-up day 21:**			
Missing values	2	3	
Physiotherapy since last follow-up, *n* (%)	21 (43.8)	16 (47.1)	0.551
ED visit since last follow-up, *n* (%)	2 (4.2)	2 (5.9)	1.000
GP visit since last follow-up, *n* (%)	14 (29.2)	16 (47.1)	0.154
Specialist visit since last follow-up, *n* (%)	7 (14.6)	3 (8.8)	0.658
Hospitalization since last follow-up, *n* (%)	2 (4.2)	0	0.632
Imaging last follow-up, *n* (%)	10 (20.8)	6 (17.6)	0.940
**Follow-up day 42:**			
Missing values	8	6	
Physiotherapy since last follow-up, *n* (%)	24 (58.5)	18 (56.2)	1.000
ED visit since last follow-up, *n* (%)	1 (2.4)	2 (6.2)	0.826
GP visit since last follow-up, *n* (%)	8 (19.5)	11 (34.4)	0.243
Specialist visit since last follow-up, *n* (%)	7 (17.1)	4 (12.5)	0.832
Hospitalization since last follow-up, *n* (%)	0	0	
Imaging last follow-up, *n* (%)	5 (12.2)	3 (9.4)	0.996

Data are reported as *n* (%). *p*-values were calculated using a significance level (alpha) of 0.05. Abbreviations: ED = emergency department, GP = general practitioner.

**Table A2:** Outcomes at follow-up day 21.

	Control (*n* = 49)	Intervention (*n* = 37)	*p*-Value
Missing values	1	3	
Oswestry Disability Index, median [IQR]	6.5 [1.00, 19.25]	8.5 [2.25, 17.5]	0.858
Oswestry Disability Index categories, *n* (%)			0.713
No disability (ODI 0–4)	18 (40.0)	13 (39.4)	
Mild disability (ODI 5–14)	19 (42.2)	17 (51.5)	
Moderate disability (ODI 15–24)	6 (13.3)	2 (6.1)	
Severe disability (ODI 25–34)	1 (2.2)	1 (3)	
Completely disabled (ODI 35–50)	1 (2.2)	0	
Pain on NRS, median [IQR]	2.00 [0.00, 4.00]	2.00 [0.00, 4.00]	0.721
Pain medication use, *n* (%)	16 (33.3)	11 (32.4)	0.690
Duration in days of painkillers intake, mean {SD}	7.52 {5.25}	9.17 {5.13}	0.252
Returned to work, *n* (%)	38 (79.2)	25 (73.5)	0.661

Data are reported as median [interquartile range], *n* (%) or mean {standard deviation}. *p*-values were calculated using a significance level (alpha) of 0.05. Abbreviations: ODI = Oswestry Disability Index, NRS = numeric rating scale.

**Table A3:** Outcomes at follow-up day 42.

	Control (*n* = 49)	Intervention (*n* = 37)	*p*-Value
Missing values	8	5	
Oswestry Disability Index, median [IQR]	4.00 [0, 16.00]	2.00 [0.75, 6.75]	0.392
Oswestry Disability Index categories, *n* (%)			0.288
No disability (ODI 0–4)	23 (56.1)	21 (65.6)	
Mild disability (ODI 5–14)	7 (17.1)	7 (21.9)	
Moderate disability (ODI 15–24)	8 (19.5)	2 (6.2)	
Severe disability (ODI 25–34)	1 (2.4)	2 (6.2)	
Completely disabled (ODI 35–50)	2 (4.9)	0	
STarT-Back Screening Tool, median [IQR]	1.00 [0, 3.00]	1.00 [0, 2.25]	0.239
STarT-Back tool risk categories, *n* (%)			0.230
Low risk	31 (75.6)	29 (90.6)	
Intermediate risk	2 (4.9)	1 (3.1)	
High risk	8 (19.5)	2 (6.2)	
Pain on NRS, median [IQR]	1 [0, 3.00]	0.5 [0, 3.00]	0.386
Pain medication use, *n* (%)	12 (29.3)	4 (12.5)	0.134
Duration in days of painkillers intake, mean {SD}	12.48 {11.75}	13.74 {8.63}	0.652
Returned to work, *n* (%)	33 (80.5)	24 (75)	0.750

Data are reported as median [interquartile range], *n* (%) or mean {standard deviation}. *p*-values were calculated using a significance level (alpha) of 0.05. Abbreviations: ODI = Oswestry Disability Index, NRS = numeric rating scale.

**Table A4:** Baseline characteristics analyzed vs. lost to follow-up (at day 0).

	Analyzed (*n* = 86)	Loss to Follow-Up (*n* = 40)
Age (in years), median [IQR]	40 [32, 52]	41 [33, 48]
Female sex, *n* (%)	35 (40.7)	11 (27.5)
BMI (in kg/m^2^), median [IQR]	25 [23, 29]	27 [23, 30]
Physical Activity Vital Sign *, median [IQR]	150 [5.5, 300]	270 [1.5, 860]
Pain medication intake before consultation, *n* (%)	60 (69.8)	31 (77.5)
ESI level, *n* (%)		
1	0	0
2	11 (12.8)	7 (17.5)
3	28 (32.6)	22 (55)
4	45 (52.3)	10 (25)
5	1 (1.2)	0 (0)
Pain on NRS (0 to 10), median [IQR]	7.5 [5, 8]	8 [6.8, 10]
STarT-Back Screening tool, median [IQR]	5 [4, 6]	5 [4, 7]
STarT-Back tool risk categories, *n* (%)		
Low risk	16 (18.6)	9 (22.5)
Intermediate risk	54 (62.8)	19 (47.5)
High risk	16 (18.6)	12 (30)
Oswestry Disability Index, median [IQR]	24 [16, 33]	29 [19, 33]
Oswestry Disability Index categories, *n* (%)		
No Disability (ODI 0–4)	2 (2.3)	1 (2.5)
Mild disability (ODI 5–14)	17 (19.8)	7 (17.5)
Moderate disability (ODI 15–24)	26 (30.2)	6 (15)
Severe disability (ODI 25–34)	24 (27.9)	19 (47.5)
Completely disabled (ODI 35–50)	17 (19.8)	7 (17.5)

Data are reported as median [interquartile range] or *n* (%). * Defined as total minutes per week of physical activity. Abbreviations: BMI = body mass index, ESI = emergency severity index, NRS = numeric rating scale, ODI = Oswestry Disability Index.
